# Towards Integrated Care for the Elderly: Exploring the Acceptability of Telemonitoring for Hypertension and Type 2 Diabetes Management

**DOI:** 10.5334/ijic.7621

**Published:** 2024-05-15

**Authors:** Matic Mihevc, Majda Mori Lukančič, Zavrnik Črt, Tina Virtič Potočnik, Marija Petek Šter, Zalika Klemenc-Ketiš, Antonija Poplas Susič

**Affiliations:** 1 Primary Healthcare Research and Development Institute, Community Health Centre Ljubljana, Metelkova ulica 9, SI-1000 Ljubljana, Slovenia; 2 Primary Healthcare Centre Trebnje, Goliev trg 3, SI-8210 Trebnje, Slovenia; 3 University of Ljubljana, Faculty of Medicine, Department of Family Medicine, Poljanski nasip 58, SI-1000 Ljubljana, Slovenia; 4 Primary Healthcare Centre Slovenj Gradec, Partizanska pot 16, SI-2380 Slovenj Gradec, Slovenia; 5 University of Maribor, Faculty of Medicine, Department of Family Medicine, Taborska ulica 8, SI-2000 Maribor, Slovenia

**Keywords:** acceptability, mobile health, telemonitoring, aged, self-management, integrated care

## Abstract

**Introduction:**

Telemonitoring has been proposed as an effective method to support integrated care for older people with hypertension and type 2 diabetes. This paper examines acceptability of telemontioring, its role in supporting integrated care, and identifies scale-up barriers.

**Methods:**

A concurrent triangulation mixed-methods study, including in-depth interviews (n = 29) and quantitative acceptability tool (n = 55) was conducted among individuals who underwent a 12-month telemonitoring routine. The research was guided by the Theoretical Framework of Acceptability. Interviews were analysed using template content analysis (TCA).

**Results:**

TCA identified seven domains of acceptability, with twenty-one subthemes influencing it positively or negatively. In the quantitative survey, acceptability was high across all seven domains with an overall score of 4.4 out of 5. Urban regions showed higher acceptability than rural regions (4.5 vs. 4.3), with rural participants perceiving initial training and participation effort as significantly more burdensome than their urban counterparts.

**Discussion:**

Patients described several instances where telemonitoring supported self-management, education, treatment, and identification elements of the integrated care package. However, there were barriers that may limit its further scale-up.

**Conclusion:**

For further scale-up, it is important to screen patients for monitoring eligibility, adapt telemonitoring devices to elderly needs, combine telemonitoring with health education, involve family members, and establish follow-up programmes.

## Introduction

Arterial hypertension (AH) and type 2 diabetes (T2D) pose significant public health challenges due to their impact on individual well-being and healthcare costs. Changes in demographics and healthcare approaches, along with projections of increased prevalence, especially in the elderly, underscore the need for effective strategies following the integrated chronic care model [1,2,3].

Primary care plays a central role in managing diseases like AH and T2D, with healthcare professionals leading disease management through screenings, treatment initiation, and ongoing guidance. Additionally, primary care serves as the main point of contact for patients, providing comprehensive health education and empowering them with self-management strategies [3,4,5].

According to WHO guidelines on integrated care and essential interventions for AH and T2D [1,2,3,4,5], an integrated care package (ICP) for patients with AH and T2D consists of six key elements: (a) early detection and diagnosis, (b) treatment in primary care services, (c) health education, (d) self-management support, (e) structured collaboration, and (f) organisation of care.

In primary care settings with high ICP implementation, such as in Slovenia [3], telemonitoring of blood pressure (BP) and blood glucose (BG) has emerged as a strategic tool to optimise and support integrated care elements for patients with AH and T2D [6,7,8,9,10,11]. Telemonitoring supports ICP elements by enabling early detection of disease deterioration, providing real-time data for treatment decisions, facilitating self-monitoring and patient education, fostering engagement, coordinating healthcare providers, and ensuring care within structured clinical pathways and treatment plans [4,6,8].

However, the sustainability of telemonitoring and its capacity to effectively support ICP elements hinge significantly on the acceptance and engagement of patients and healthcare workers [12,13,14,15]. Previous studies have already established the good acceptability of telemonitoring among patients and healthcare workers [15,16,17,18,19], even within the Slovenian setting [6,20,21]. However, despite the recognised importance of acceptability, previous research has struggled to define acceptability and used its proxies, such as satisfaction, dropout rates, recruitment rates, adherence to protocol or adverse events instead of the more comprehensive Theoretical Framework of Acceptability (TFA) definition [14].

According to the TFA [14], acceptability of healthcare intervention is a seven-domain-construct that “reflects the extent to which people receiving healthcare intervention consider it appropriate, based on anticipated or experimental cognitive and emotional responses to the intervention”.

Furthermore, there is a gap in our understanding regarding acceptability of telemonitoring in older patients, who represent a complex and rapidly growing population [2,4]. In older patients, telemonitoring may elicit different cognitive and emotional responses due to factors such as cognitive impairment, multiple chronic conditions, reliance on environmental support, hesitancy toward adopting modern technologies, and physical limitations such as reduced vision or motor skills [22].

To address this knowledge gap, we conducted a mixed-methods study informed by TFA to (a) assess the acceptability of telemonitoring among older patients in urban and rural regions of Slovenia, (b) identify its role in supporting the ICP, and (c) identify barriers that may limit its further scale-up within the integrated care model for patients with AH and T2D in primary care setting.

## Methods

### Study Design

We conducted a concurrent triangulation mixed-methods study from March 2022 to June 2023. The study was nested within a larger parent study investigating the clinical effectiveness and cost structure of telemonitoring in patients with AH and T2D [4,23].

### Ethic and Consent

The study was approved by the Medical Ethics Committee of the Republic of Slovenia (0120-219/2019/4) and was registered in the ISRCTN registry (https://doi.org/10.1186/ISRCTN31471852). The study aligned with the Declaration of Helsinki. Before participation, patients were informed about the aims of the study and signed an informed consent form.

### Study Setting

The study took place in three primary healthcare centres (PHCs) in Slovenia, each representing different development contexts. PHC Ljubljana, serving about 300,000 residents, reflected an urban profile with a gross domestic product (GDP) of 108% of the EU mean in 2021. In contrast, PHC Trebnje and PHC Slovenj Gradec, catering to around 50,000 residents in Eastern Slovenia, exemplified rural settings with a GDP of 74% of the EU mean in 2021 [24].

### Sampling Strategy

The original randomised controlled study [4] was conducted over two periods: round 1 from March 2021 to April 2022 and round 2 from May 2022 to June 2023. During this time, a total of 128 patients were randomly assigned to either the telemonitoring group (n = 64) or the standard care group (n = 64). The necessity for two rounds arose due to limited telemonitoring equipment availability. Following a 12-month follow-up period, 55 out of 64 patients (85.9%) completed the telemonitoring intervention. Reasons for early dropout included difficulties in using the telemonitoring equipment correctly (n = 7), psychological burden (n = 1), and the death of a spouse (n = 1).

Inclusion criteria were: (a) age ≥65 years, (b) comorbidity of AH and T2D for at least one year, (c) ability to use telemonitoring equipment. Exclusion criteria were: (a) T2D requiring insulin treatment at inclusion, (b) type 1 diabetes, (c) cognitive impairment, and (d) inability to use telemonitoring devices for various reasons.

For this mixed-methods study, we initially recruited patients from round 1 who were assigned to the telemonitoring intervention (n = 30). All 30 patients agreed to participate in the quantitative acceptability survey, while one declined to participate in the semi-structured interviews (29/30). After the first round, we achieved data saturation in the qualitative segment. Nevertheless, to underpin the reliability of our quantitative findings, we extended our quantitative analysis to encompass participants from round 2 as well, culminating in a total of 55 participants.

### Integrated Care Model

In Slovenia, a team made up of a general practitioner (GP) and a registered nurse provides integrated care for patients with AH and T2D at the primary care level [3,4,7]. This team conducts annual check-ups on patients, with the physician conducting physical examinations, requesting laboratory tests, assessing disease control, and determining if adjustments to treatment are necessary. The registered nurse, on the other hand, screens patients for complications related to AH and T2D, evaluates their psychosocial status, educates them on non-pharmacological measures, and refers them to health promotion activities at the health education centre. Standard protocols for diagnosis and treatment, health education, and guidelines for collaboration among various healthcare providers are used to ensure coordinated care for patients. Although the implementation of ICP in Slovenia is high, improvements are still needed in the aspects related to self-management and structured collaboration [3].

### Telemonitoring Intervention

To improve self-management support and structured collaboration within primary care units in Slovenia, we introduced a telemonitoring intervention alongside standard care [4]. Participants were equipped with a telemonitoring package comprising a smartphone and monitors for BP and BG, which they used over a 12-month period. These devices transmitted data to a mobile application via Bluetooth, serving as a central hub, which then transferred the measurement results to the telemedicine cloud platform using a 4G or 5G mobile standard. Upon enrolment, participants underwent a one-hour training session led by a registered nurse. This one-on-one session covered the telemonitoring regimen and provided instructions on how to use the telemonitoring equipment.

Under the supervision of a GP, the telemedicine centre monitored recorded values and facilitated communication between other GPs and enrolled patients on weekdays. Throughout the 12-month period, participants measured their BP twice weekly and their BG once monthly. The transmitted values were colour-coded to indicate their status: green for normal, yellow for slightly off-target, and red for significantly deviated values. A green status indicated no changes were necessary, while yellow prompted adjustments to the measurement protocol, such as a 7-day BP profile with morning and evening measurements for AH or a one-day BG profile with six measurements for T2D. In cases of red status, immediate teleconsultation with a GP or further examination was required.

Patients were managed according to recent guidelines [25], aiming for home BP <135/85 mmHg, fasting BG <7 mmol/l and postprandial BG <10 mmol/l, with treatment goals adjusted for the elderly.

### Data Collection

#### Theoretical framework

**Figure 1:**
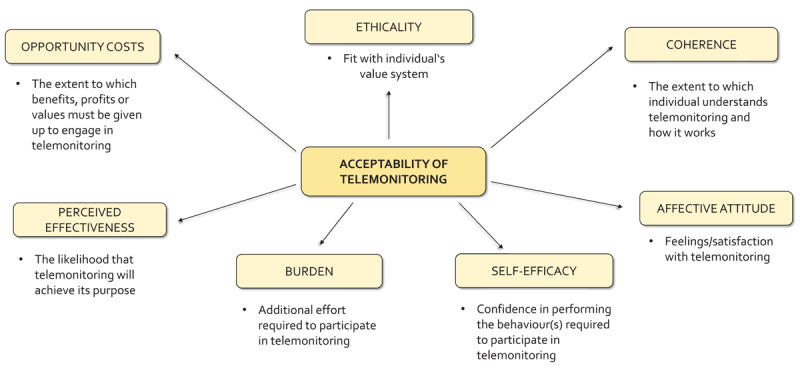
TFA domains in the context of telemonitoring.

The semi-structured interviews and acceptability tool were informed by the TFA [14]. The TFA describes acceptability as a multi-faceted construct consisting of the seven domains explained in Figure 1, which we adapted to measure retrospective acceptability of the telemonitoring and estimate its role in the integrated care model.

#### Demographic and clinical profile

Participants completed a structured questionnaire detailing demographic and clinical characteristics, including age, gender, education, marital status, smoking, and duration of AH and T2D. We obtained data on systolic and diastolic BP, HbA1c levels, and body mass index from medical records and the telemonitoring platform. To assess the time involved in telemonitoring, we extracted data from the platform on adherence to BP and BG protocols, teleconsultation frequency per year, and annual BP and BG measurement time. Further details are available in the cost structure article [23].

#### Semi-structured interviews

Semi-structured interviews were conducted face-to-face and lasted on average 25 minutes (range 20–35 minutes). They took place between April-June 2022 at three locations: PHC Ljubljana, PHC Trebnje, and PHC Slovenj Gradec. The topic guide (Appendix 1) included topics related to health context, recruitment process, coping with technology, telemonitoring routine, change in self-management skills, impact on mental health, and change in patient-doctor relationship and chronic care. The topic guide was pilot tested with 5 patients and later checked for comprehensibility.

Two researchers (MM, MML) conducted the interviews, both experienced in qualitative content analysis. MM is a GP and conducted 21/29 (72.4%) interviews. MML is a nurse and conducted 8/29 (27.6%) interviews. The patients met MML once in person at the training 12 months before the interview (mean time 82 minutes), whereas they had not met MM in person before. However, 22/29 (75.8%) had been in telephone contact with MM during telemonitoring (mean time 38 minutes). The interviews were audio recorded and transcribed verbatim by MM and MML, and the sample size was based on saturation process. The patients were aware of the interviewers’ profession, personal goals, and reasons for doing the research. No compensation was given for patient’s time. There were no non-participants observing the interviews and there were no transcripts returned to participants for comments.

#### Quantitative acceptability tool

Acceptability tool (Appendix 2) was developed to assess each of the seven constructs outlined in the TFA framework quantitatively. For each construct, one to three carefully chosen statements were provided, and participants rated them on a five-point Likert scale from 1 (strongly disagree) to 5 (strongly agree). These statements were selected based on insights from three sources: (a) a theory-informed questionnaire recommended by the original author of the TFA framework [26], (b) findings from earlier studies that employed TFA for quantitatively assessing acceptability [27], and (c) insights from previous pilot and feasibility studies on telemonitoring in Slovenia [6,20,21]. The content validity of the scale was evaluated by six researchers in the areas of primary care, diabetes education and management, and implementation science. The Cronbach’s alpha of this new instrument was 0.86.

### Data Analysis

#### Qualitative analysis

Qualitative analysis was conducted using QSR NVivo software (version 1.6.1). The analysis began with a top-down approach using Template Content Analysis [28] with seven constructs from the TFA as a guide. Two coders (MM, ČZ) independently coded the first 10 interviews using the TFA template with the option to add codes inductively derived from the interviews if needed. After coding the first 10 interviews, an inter-coder meeting was held to compare codebooks and resolve any differences. A third researcher (MML) was consulted if necessary. Once the preliminary codebook was established, the remaining interviews were coded. Upon coding 25 interviews, it was noted that no new themes or subthemes emerged, suggesting a level of data saturation. However, to confirm this observation and ensure comprehensive data analysis, the coding process extended to encompass all 29 conducted interviews. This deliberate approach aimed to validate the saturation point and enhance the credibility of the study findings through a thorough examination of the entire dataset. After all interviews were coded, a group of six researchers reviewed both codebooks and completed the analysis.

#### Quantitative analysis

The distribution characteristics of the sample were initially assessed using the Shapiro-Wilks test. This revealed a normal distribution of the clinical characteristics and a non-normal distribution of the results of the quantitative acceptability tool. Numerical variables were summarised with mean and standard deviation in the case of a symmetrical distribution or with median and minimum and maximum values in the case of an asymmetrical distribution. Categorical variables were summarised with frequencies and percentages. To analyse differences between urban and rural regions in the assessment of the individual TFA constructs, the Mann-Whitney U-test was used for individual statements and the independent samples t-test for combined values. The statistical analysis was performed using IBM SPSS Statistics (version 25.0), with a p-value of less than 0.05 being considered statistically significant.

#### Data triangulation

**Figure 2:**
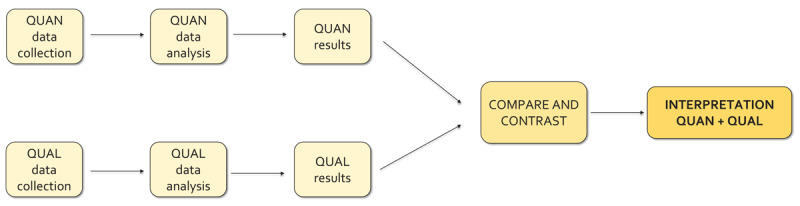
Convergence model used for data triangulation.

After completing both analyses, we applied a convergence model of data triangulation to our data [29]. This model involves gathering and examining quantitative and qualitative data on a phenomenon separately and then combining the results through comparison in the interpretation phase (Figure 2). The aim was to validate the results, improve reliability, and gain a more comprehensive understanding of telemonitoring in terms of its acceptability, the components of the ICP it supported, and the barriers to its further scale-up within the integrated care model.

## Results

### Participant characteristics

The study included 55 participants in the quantitative survey and 29 in the qualitative study, representing urban and rural regions of Slovenia with different demographic characteristics (Table 1). Despite challenges in managing their cardiometabolic risk factors, which were comparable to the Slovenian population of older people with T2D [2], participants showed high adherence in monitoring BP and BG levels, spending an average of 14–15 hours per year on monitoring.

**Table 1:** Characteristics of the included participants.


CHARACTERISTIC	QUALITATIVE STUDY (n = 29)	QUANTITATIVE SURVEY (n = 55)

**SOCIO-DEMOGRAPHIC PROFILE**		

**Mean age (years, SD)**	70.6 (5.1)	70.6 (4.4)

**Sex**		

Male (%)	58.6	61.8

Female (%)	41.4	38.2

**Highest education achieved**		

Primary school (%)	10.3	12.7

Vocational school (%)	58.6	60.0

High school graduate (%)	20.7	18.2

Bachelor’s degree (%)	6.9	5.5

Master’s degree (%)	3.4	3.6

**Region**		

Urban (%)	69.0	49.1

Rural (%)	31.0	50.9

**Marital status**		

Married (%)	69.0	74.5

Divorced (%)	3.4	3.6

Widowed (%)	20.7	18.2

Single (%)	6.9	3.6

**Smoking**		

Yes (%)	20.7	16.4

No (%)	79.3	83.6

**CLINICAL CHARACTERISTICS**		

Mean duration of type 2 diabetes (years, SD)	10.6 (6.6)	9.8 (6.4)

Mean duration of hypertension (years, SD)	15.8 (10.9)	13.9 (10.7)

Mean systolic blood pressure (mmHg, SD)	143.0 (12.9)	136.4 (14.6)

Mean diastolic blood pressure (mmHg, SD)	76.3 (7.7)	75.8 (6.9)

Mean HbA1c (%, SD)	7.3 (1.5)	7.3 (1.2)

Mean body mass index (kg/m^2^, SD)	29.6 (4.4)	30.2 (4.6)

**TELEMONITORING ROUTINE**		

Mean adherence to BP protocol (%, SD)	142.6 (50.4)	146.5 (70.5)

Mean adherence to BG protocol (%, SD)	269.4 (183.2)	252.8 (180.6)

Mean number of teleconsultations per year (n, SD)	2.5 (1.7)	2.5 (1.6)

Mean annual time to perform BP and BG measurements (min, SD)	878 (231)	885 (380)


**Legend:** SD – standard deviation, BP – blood pressure, BG – blood glucose.

### Qualitative Results

During the qualitative analysis, we delineated seven overarching themes and twenty-one subthemes, as detailed in Table 2. These themes are structured in alignment with the TFA, while the subthemes are arranged based on their emergence sequence during the analysis process. Additionally, we assessed the influence of each subtheme on acceptability, indicating whether it had a positive influence (⊕) by facilitating acceptability or a negative influence (⊖) by impeding it, according to the views of participants.

**Table 2:** Coding tree representing the main themes and subthemes identified through qualitative data analysis and their anticipated influence on acceptability.


THEME	SUBTHEME	INFLUENCE ON ACCEPTABILITY

**Theme 1:** Affective attitude	1.1 Anticipatory anxiety in case of technical problems	⊖

	1.2 Attitude towards measurement protocol	⊖

	1.3 Reassuring effect of normal readings	⊕

	1.4 Role of patient-doctor relationship	⊕

**Theme 2:** Burden	2.1 Burden of measurement routine	⊖

	2.2 Inexperience with modern technologies	⊖

	2.3 Technical problems	⊖

	2.4 Burden of underlying diseases	⊖

**Theme 3:** Perceived effectiveness	3.1 Behavioural changes	⊕

	3.2 Understanding relationship between lifestyle and glycaemic control	⊕

	3.3 Faster modification of therapy	⊕

	3.4 Lack of integration into the public healthcare system	⊖

	3.5 Regular response from telemedicine centre	⊕

**Theme 4:** Ethicality	4.1 Limited integration into patient environment	⊖

	4.2 Partner or family support	⊕

**Theme 5:** Coherence	5.1 Internalisation of benefits	⊕

	5.2 Education training	⊕

**Theme 6**: Self-efficacy	6.1 Confidence in self-management skills	⊕

	6.2 Previous experience with self-management of diabetes	⊕

**Theme 7:** Opportunity costs	7.1 Burden on family members	⊖

	7.2 Taking measurements during holidays	⊖


#### Theme 1: Affective attitude

Participants reported overall positive experiences with some feeling reassured by normal readings and others finding telemonitoring frustrating or causing anticipatory anxiety, especially when there were technical problems. The latter affected reliability of measurements. Most did not find the measurement routine burdensome and expressed satisfaction with the delivery of the intervention and “benign” policy control. A good prior relationship with their GP was important to participants and influenced their decision for participation. However, they also expressed fear that non-participation would affect this relationship but did not report coercion to participate.

“When something did not go my way, I was more nervous. I am the type of person who gets upset quickly and then everything went up – blood pressure and blood glucose.” (Participant 6, female, 70 years, urban)

“I had no reservations. I was very happy and delighted that my GP invited me to this programme. He knows what is good for me.” (Participant 1, female, 73 years, urban)

#### Theme 2: Burden

Most participants in telemonitoring intervention found it easy to participate and did not find it burdensome. Participants experienced technical burdens when there were problems with forgotten passwords, inability to send readings, and issues with the use of smartphone and BG monitor. They also faced underlying medical conditions that made it difficult to improve their lifestyle and follow the routine. Despite these challenges, most felt obliged to continue due to the positive impact on patient-doctor relationships.

“I think it should be easier. I had problems with my password because it contained upper- and lower-case letters, so I accidently blocked the application. Then there were problems with my phone, and I had to take it twice to my GP.” (Participant 23, male, 71 years, rural)

“I have been getting a little sick of it lately. It is necessary to measure blood pressure twice a week and then not to forget blood glucose. I had put the devices on the table before I went to bed so that I did not forget to take the measurements in the morning.” (Participant 2, female, 74 years, urban)

#### Theme 3: Perceived effectiveness

Participants had high confidence in the effectiveness of telemonitoring and reported that it helped them better understand their disease and improve their health. They also felt more experienced in self-managing their disease. Participants reported changing their behaviour based on their measurements and appreciated regular feedback from the telemedicine centre. They suggested wider implementation of telemonitoring in the public healthcare system to reach more people and address financing issues.

“I felt good, but the readings were not and that bothered me. When I saw blood glucose values jumping up and down, it shook me up a bit. Now, I even use a health app to check what I am eating.” (Participant 7, female, 67 years, rural)

“It was nice that you regularly checked the measurements and answered questions. I did not think that would happen. But then I saw that the answer came quickly, and I said – great, at least someone is checking it regularly.” (Participant 7, female, 67 years, rural)

#### Theme 4: Ethicality

Telemonitoring was in line with the life beliefs and values of patients. Participants had diverse backgrounds and responsibilities, including being widowed and living alone, caring for a seriously ill partner, and caring for grandchildren, which sometimes hindered regular monitoring. The main motivators for some participants were their partners and children.

“I have a sick husband and it is very stressful for me. I sleep very badly. I am afraid I will not be able to take care of myself and my husband.” (Participant 2, female, 74 years, urban)

“I participated because my family promised to help. My daughter was very happy that I stick to something and regularly checked the messages. They even bought me new monitors for my birthday, and I plan to continue with measuring routine”. (Participant 26, female, 82 years, rural)

#### Theme 5: Coherence

Participants had a good understanding of telemonitoring and appreciated its benefits, such as free monitors and improved health outcomes. Training on device use was sufficient, but some first-time learners found the instructions too quick and came back for further guidance. They appreciated that all instructions were included in the user manual.

“I included because it seemed wise for my health. Because I was convinced that it was also important to remind myself to watch what I eat. Otherwise, I would not be so careful with my diet and medication.” (Participant 25, female, 75 years, rural)

#### Theme 6: Self-efficacy

Participants felt confident in using telemonitoring skills in daily life and had routines for regular measurements. They felt more responsible and confident in responding to changes in BP and BG and had strategies in place for control and knew when to consult their GP. Patients with previous experience of managing T2D learned that they could control their disease in the long term and felt more confident in self-managing it.

“I have diabetes and hypertension for the last 30 years. I follow a strict diet and exercise. The last year has shown me that I am able to control both diseases in the long term.” (Participant 2, female, 74 years, urban)

#### Theme 7: Opportunity costs

Participants felt that telemonitoring did not impact their hobbies, family, or friends. They had issues maintaining telemonitoring routine on vacation and often left measurements behind. There was an additional burden placed on family members, who often had to help with measurements.

“I have also taken devices with me on holiday. I took it to the spa. There, I accidentally deleted the phone app and had to call technical support.” (Participant 3, male, 71 years, urban)

“What do I know? I did not know anything, and I had a hard time waiting for my daughter to get off work. I am old and forgetful. That is why my daughter did it.” (Participant 26, female, 82 years, rural)

### Quantitative Results

The overall acceptability of telemonitoring was high (4.4 out of 5) in the quantitative survey, with favourably ratings in all seven TFA domains (Table 3). However, the overall acceptability was higher in urban regions compared to the rural regions (4.5 vs. 4.3). Participants in rural regions perceived initial training and participation effort as significantly more burdensome than their urban counterparts. They found that telemonitoring was in lower agreement with their beliefs and values and had a lower understanding of the objectives of telemonitoring. On the other hand, they felt that after 12 months of telemonitoring they had gained more knowledge and understanding of their disease than the participants from the urban regions.

**Table 3:** Quantitative ratings of acceptability domains.


DIMENSION	URBAN REGION(n = 27), MEDIAN(min, max)	RURAL REGION(n = 28), MEDIAN (min, max)	COMBINED(n = 55), MEDIAN (min, max)	p

**1 AFFECTIVE ATTITUDE**				

1.1 Satisfaction with opportunity to participate	5 (4, 5)	5 (3, 5)	5 (3, 5)	0.150

1.2 Satisfaction with telemonitoring course	5 (4, 5)	5 (2, 5)	5 (2, 5)	0.471

**2 BURDEN**				

2.1 Little extra time required to participate	5 (4, 5)	4 (1, 5)	5 (2, 5)	0.182

2.2 Much extra time required to learn how to use telemonitoring equipment (R)	4.5 (1, 5)	4 (1, 5)	4 (1, 5)	0.009

2.3 Much extra effort needed to participate (R)	5 (1, 5)	4 (1, 5)	4 (1, 5)	0.004

**3 PERCEIVED EFFECTIVENESS**				

3.1 Improved knowledge and understanding of diseases	4.5 (3, 5)	5 (4, 5)	5 (3, 5)	0.380

3.2 Improved health	5 (4, 5)	4 (3, 5)	4 (3, 5)	0.362

3.3 Gained experience with self-management skills	4 (3, 5)	4 (1, 5)	4 (1, 5)	0.786

**4 ETHICALITY**				

4.1 Agreement with individual’s beliefs and values	5 (4, 5)	4 (1, 5)	4 (1, 5)	0.101

**5 COHERENCE**				

5.1 Adequate data on content and process of telemonitoring	4.5 (3, 5)	4 (3, 5)	4 (3, 5)	0.716

5.2 Good understanding of objectives of telemonitoring	5 (4, 5)	4 (3, 5)	4.5 (3, 5)	0.298

**6 SELF-EFFICACY**				

6.1 Confidence in applying the skills learned in daily life	4 (3, 5)	4 (4, 5)	4 (1, 5)	0.557

**7 OPPORTUNITY COSTS**				

7.1 Less time for family and friends (R)	5 (1, 5)	5 (2, 5)	5 (1, 5)	0.921

7.2 Less time for hobbies (R)	5 (3, 5)	5 (1, 5)	5 (1, 5)	0.905

**MEAN COMBINED SCORES** **(95% CI)**	**4.5** **(4.4–4.7)**	**4.3** **(4.1–4.5)**	**4.4** **(4.3–4.5)**	**0.066**


**Legend:** R – reversed scores are shown.

## Discussion

### Principal Findings and Comparison with the Existing Literature

The results of the study showed that older people with AH and T2D found telemonitoring to be highly acceptable in all seven TFA domains and provided examples of how telemonitoring supported the ICP elements. However, there were discrepancies between the measured acceptability through the quantitative acceptability tool and the barriers identified through the qualitative content analysis.

Telemonitoring systems are generally well accepted by patients with chronic diseases, such as heart failure [15], chronic obstructive pulmonary disease [16], AH [17], and T2D [18,19]. However, previous studies relied on proxies of acceptability such as satisfaction [15,16,18,19], perceived benefits [18], or intention to use in the future [16,17,19], making a direct comparison with TFA difficult. Furthermore, high acceptability does not necessarily guarantee an effective intervention [13], and there are other factors that influence scalability and sustainability of the intervention [12,13].

Patients mentioned several facilitating factors that influenced acceptability and supported the ICP components. A positive patient-doctor relationship, perceived benefits, and previous positive experiences with self-management or the use of modern technologies were important motivating factors at the start of the intervention. Throughout the telemonitoring process, good family support, successful adjustment to their environment and beliefs, reassurance from normal clinical parameters, and regular feedback from the telemedicine centre emerged as important motivating factors for patients. Most of these factors were also identified in previous studies and should be reinforced during telemonitoring [12,15,16,17,18,19].

On the other hand, patients described factors that might hinder these processes, such as technical difficulties, negative attitudes towards measurement routines, anxiety, taking measurements during holidays, and additional burden on family members. Personal beliefs and financial resources were also found to be potential barriers in previous studies [12,19,20]. Additionally, some studies have pointed to the risk of focusing solely on monitoring health parameters rather than the overall health of patients [30].

Participants in rural regions perceived initial training and participation effort as significantly more burdensome than their urban counterparts. They found that telemonitoring was in lower agreement with their beliefs and values and had a lower understanding of the objectives of telemonitoring. On the other hand, they felt that after 12 months of telemonitoring they had gained more knowledge and understanding of their disease than participants from the urban region. This discrepancy could be explained by the Diffusion of Innovation Theory [31], which suggests that the adoption of new health technologies requires changes in healthcare organisations, and that adoption is slower in rural areas because there are fewer early adopters, less support from healthcare leaders, and limitations in technological infrastructure than in urban areas [32].

Ultimately, patients described several ways in which telemonitoring supported the self-management, education, treatment, and identification components of the ICP [1,3,8]. Regular self-monitoring and timely feedback from medical staff facilitated the education process, and patients learned how non-pharmacological measures improve glycaemic control and were motivated to achieve better health outcomes [33]. However, this approach was less effective in controlling BP, where levels of BP were less stable, and patients were more reliant on treatment modification and stress management [17]. In terms of early identification and treatment, patients were provided with monitors and clinical protocols that allowed them to detect deterioration in their health earlier than usual and adjust their AH/T2D treatment without having to see their doctor [12].

### Implications for Research and Practice

Despite good overall acceptability, our study identified several scale-up barriers in the areas of screening, technical support, integration of telemonitoring into patient homes, education, and follow-up programmes that need to be addressed before scaling up telemonitoring in integrated care models for older patients.

#### Screening for appropriate patients

Telemonitoring is beneficial for older patients [9,10,21,23], but external surveillance routines and modern technology may burden them [6,12,21], leading to early dropouts. To prevent this, patients should be screened for technological literacy, communication preferences, motivation, and expectations before inclusion [6,20]. Screening tools such as the Mobile Device Proficiency Questionnaire (MDPQ-16) [34], Adapted Technology Acceptance Model questionnaire [34] or anticipatory acceptability tool [35] could be used for this purpose. Patients who perform poorly may need additional training or may be better suited for simpler, paper-based methods.

#### Adaptation of telemonitoring equipment to elderly needs

In our study, patients reported encountering various technical challenges, including difficulties with the touch screen interface, forgotten passwords, low battery life, and pairing medical monitors. To tackle these issues, telemonitoring solutions should incorporate devices with larger screens, such as tablets, and features like well-labelled icons, a spacious keyboard, voice control commands, and alternative methods of accessing the application, such as fingerprint or lock patterns [22,23]. Additionally, replacing fingerstick BG meters with subcutaneous continuous glucose monitoring systems could improve acceptability, especially in individuals using insulin therapy [36].

#### Engaging family members in remote care models

In our study, participants cited family members as important sources of motivation and assistance when encountering technical challenges. However, they lacked familiarity with the telemonitoring equipment and monitoring routine. Previous studies have shown that when involving family members in remote care models, they can become integral parts of the patient’s treatment plan, facilitating monitoring, and addressing their needs [21,37,38]. Nevertheless, research suggests that this approach may also introduce additional burden and anxiety [21,38].

#### Combining telemonitoring with health education interventions

To promote patient empowerment and improve lifestyle risk factors control, telemonitoring should be paired with health education interventions that help patients learn about their disease and how to manage their own care, including lifestyle changes. This approach has been shown to lead to better outcomes in the long-term [9,10].

#### Setting up follow-up programmes

There is currently uncertainty about whether telemonitoring is cost-effective for patients with AH or T2D, leading to many short-term pilot studies [9,10,23]. Despite high perceived effectiveness and self-efficacy, these effects may diminish after two years [9,10]. To sustain telemonitoring benefits and maintain continuity of care, we suggest including follow-up meetings with registered nurse in future interventions to review clinical outcomes and non-pharmacological adherence over the long term.

### Strengths and Limitations

The strength of this study lies in its mixed methods approach to investigating the acceptability of telemonitoring, embedded in a larger randomised controlled trial, and in diverse population from different centres and regions. In addition, telemonitoring was also examined from the perspective of how it supports the integrated care elements in older people with AH and T2D. However, the small sample size and inclusion of only motivated participants may limit the generalisability of the findings. Although the initial response rate to participate in the intervention was high (128/160, 80%), potentially mitigating the impact of this limitation. In addition, we did not return the transcripts to participants for comments or corrections, which could limit the validity of the interviews. However, we attempted to limit this limitation using the convergence model of data triangulation. Finally, we only considered the views of older participants, who have rarely been studied before, whereas views of the healthcare workers involved in the telemonitoring process could also be beneficial.

## Conclusion

Our research has improved our understanding of the extent to which older people with AH and T2D accept telemonitoring and how it supports the various components of the ICP. While participants reported high levels of acceptability in the quantitative survey, a deeper insight into their experiences revealed important barriers to further scale-up in the areas of screening, technical support, integration of telemonitoring into patient homes, education, and follow-up programmes. Ultimately, patients described several ways in which telemonitoring supported the self-management, education, treatment, and identification components of the ICP. However, further research should also explore the views of other stakeholders involved in the telemonitoring process to fully assess the potential of telemonitoring in supporting the ICP, particularly the perspectives of structured collaboration and organisation of care.

## Additional File

The additional file for this article can be found as follows:

10.5334/ijic.7621.s1 Appendices. Appendices 1 and 2.
