# Supplementary material for: Towards Integrated Care for the Elderly: Exploring the Acceptability of Telemonitoring for Hypertension and Type 2 Diabetes Management
Source: Int J Integr Care. 2024 May 15;24(2):16. doi: 10.5334/ijic.7621 (PMC11100527; doi:10.5334/ijic.7621)
Supplement: Appendices. — Appendices 1 and 2. [file ijic-24-2-7621-s1.pdf]

**Appendix 1: GUIDE FOR CONDUCTING SEMI-STRUCTURED INTERVIEWS**

*Dear [Participant],*

*Today we will be discussing your experiences and perspectives regarding telemonitoring. We are interested in your opinions and specific experiences as a participant in a study involving telemonitoring. Please provide detailed responses to the questions and share your honest opinions.*

1. *What is your opinion on telemonitoring? What advantages and drawbacks have you noticed?*
2. *How did your GP manage your hypertension and diabetes before your participation in the study?*
3. *Why did you decide to participate in the study [telemonitoring]?*
4. *How did you experience the training on the use of monitoring equipment with the nurse educator and what were your feelings in the first month of study participation?*
5. *How did you cope with using monitoring equipment and what are your experiences with technical support during telemonitoring?*
6. *What are your experiences and opinions about measurement protocol?*
7. *How has telemonitoring affected communication with your GP and other healthcare professionals?*
8. *How has telemonitoring affected your life, daily activities, and mood?*
9. *How has telemonitoring influenced your understanding of your health conditions and your ability to manage them on your own?*
10. *How has your relationship with your GP changed due to participation in the study?*
11. *How has the role of family members in monitoring your diseases changed due to participation in the study?*

*Is there anything else you would like to add/praise/criticise that we have not discussed regarding telemonitoring?*

**Appendix 2: QUANTITATIVE ACCEPTABILITY TOOL**

*With the following tool, we aim to assess the acceptability of telemonitoring. Please rate your agreement with the statements below on a scale from 1 (strongly disagree) to 5 (strongly agree).*

**1 AFFECTIVE ATTITUDE**

1.1 I am satisfied to have had the opportunity to participate in telemonitoring.

| | | | | |
|---|---|---|---|---|
| 1<br>Strongly disagree | 2<br>Disagree | 3<br>Neither agree nor disagree | 4<br>Agree | 5<br>Strongly agree |

1.2 I am satisfied with the course of telemonitoring.

| | | | | |
|---|---|---|---|---|
| 1<br>Strongly disagree | 2<br>Disagree | 3<br>Neither agree nor disagree | 4<br>Agree | 5<br>Strongly agree |

**2 BURDEN**

2.1 It was easy to find time to participate in telemonitoring.

| | | | | |
|---|---|---|---|---|
| 1<br>Strongly disagree | 2<br>Disagree | 3<br>Neither agree nor disagree | 4<br>Agree | 5<br>Strongly agree |

2.2 Training on the use of telemonitoring equipment took too much extra time.

| | | | | |
|---|---|---|---|---|
| 1<br>Strongly disagree | 2<br>Disagree | 3<br>Neither agree nor disagree | 4<br>Agree | 5<br>Strongly agree |

2.3 I had to put in a lot of effort to participate in telemonitoring.

| | | | | |
|---|---|---|---|---|
| 1<br>Strongly disagree | 2<br>Disagree | 3<br>Neither agree nor disagree | 4<br>Agree | 5<br>Strongly agree |

**3 PERCEIVED EFFECTIVENESS**

3.1 Telemonitoring helped me to improve knowledge and better understand my disease.

| | | | | |
|---|---|---|---|---|
| 1<br>Strongly disagree | 2<br>Disagree | 3<br>Neither agree nor disagree | 4<br>Agree | 5<br>Strongly agree |

3.2 I believe that participating in telemontioring contributed to improving my health.

| | | | | |
|---|---|---|---|---|
| 1<br>Strongly disagree | 2<br>Disagree | 3<br>Neither agree nor disagree | 4<br>Agree | 5<br>Strongly agree |

3.3 Participating in telemonitoring has made me feel more experienced in self-management and self-care of my disease.

| | | | | |
|---|---|---|---|---|
| 1<br>Strongly disagree | 2<br>Disagree | 3<br>Neither agree nor disagree | 4<br>Agree | 5<br>Strongly agree |

**4 ETHICALITY**

4.1 I believe telemonitoring remote care model aligns well with my life beliefs and values.

| | | | | |
|---|---|---|---|---|
| 1<br>Strongly disagree | 2<br>Disagree | 3<br>Neither agree nor disagree | 4<br>Agree | 5<br>Strongly agree |

**5 COHERENCE**

5.1 I believe I received enough information about the content and process of telemonitoring.

| | | | | |
|---|---|---|---|---|
| 1<br>Strongly disagree | 2<br>Disagree | 3<br>Neither agree nor disagree | 4<br>Agree | 5<br>Strongly agree |

5.2 I believe it is easy to understand what the main objectives of telemonitoring are.

| | | | | |
|---|---|---|---|---|
| 1<br>Strongly disagree | 2<br>Disagree | 3<br>Neither agree nor disagree | 4<br>Agree | 5<br>Strongly agree |

**6 SELF-EFFICACY**

6.1 I am confident that I will be able to apply the skills learned through telemonitoring in everyday life.

| | | | | |
|---|---|---|---|---|
| 1<br>Strongly disagree | 2<br>Disagree | 3<br>Neither agree nor disagree | 4<br>Agree | 5<br>Strongly agree |

**7 OPPORTUNITY COSTS**

7.1 Because of participating in telemonitoring, I have spent less time with my family and friends.

| | | | | |
|---|---|---|---|---|
| 1<br>Strongly disagree | 2<br>Disagree | 3<br>Neither agree nor disagree | 4<br>Agree | 5<br>Strongly agree |

7.2 Because of participating in telemonitoring, I had less time for my hobbies.

| | | | | |
|---|---|---|---|---|
| 1<br>Strongly disagree | 2<br>Disagree | 3<br>Neither agree nor disagree | 4<br>Agree | 5<br>Strongly agree |
